# Medical Associations

**Published:** 1879-02

**Authors:** 


### Editorial.

### MEDICAL ASSOCIATIONS.

The season for State and National assemblies of medical men is approaching, and we shall look for lively discussions on the subject of malignant epidemics which, in Asia and America, have of late provod so destructive of life.

The State Association, which meets the third Wednesday in April, at Rome, will, doubtless, have pretty full attendance. Those familiar with the nature and peculiarities of epidemics, particularly those which destroy our own people, will, we hope, present at this meeting facts for the consideration of the profession, practical and useful. Each member receives information from the labors of others, and is due his mite at any rate to the general cause of advancement in practical knowledge. If all go solely to learn from others there will be no one to learn from. These meetings are intended to receive contributions, and every member can afford something. It is not particularly important that he present a learned theoretical dissertation. A fact, one fact, established beyond dispute, by thorough investigation and experiment, adds more to the advancement of medical science than a dozen fancy theoretrical papers, and will be more highly appreciated by those possessing judgment and practical sense.

The American Medical Association, which meets in Atlanta on the 6th of May, will give to this portion of the South a treat with which we have not before been favored. Many who have not heretofore had an opportunity to be present at and contribute through this medium will, doubtless, be present on this occasion. The most active, learned and useful American medical men often attend the meetings of this association, and those living near, who do not wish to tax themselves with the labor and loss of time to contribute, should not fail to witness the discussions and deliberations of the meeting.

